# The Association of Serum IL-10 Levels with the Disease Activity in Systemic-Onset Juvenile Idiopathic Arthritis Patients

**DOI:** 10.1155/2021/6650928

**Published:** 2021-03-22

**Authors:** Yu Peng, Xiaohui Liu, Zhao Duan, Junkai Duan, Yulan Zhou

**Affiliations:** ^1^Jiangxi Province Children's Hospital, Nanchang, 330006 Jiangxi, China; ^2^The Affiliated Children's Hospital of Nanchang University, Nanchang, 330006 Jiangxi, China; ^3^The First Affiliated Hospital of Nanchang University, Nanchang, 330006 Jiangxi, China

## Abstract

**Objectives:**

Interleukin-10 (IL-10) has been suggested as a biomarker of disease activity in patients with adult-onset Still's disease (AOSD). In this study, we evaluated the serum IL-10 levels and investigated its clinical relevance in systemic-onset juvenile idiopathic arthritis (SoJIA).

**Methods:**

IL-10 levels were determined in 21 patients diagnosed with SoJIA and 35 patients with fever diseases which were suspected as SoJIA, and IL-10 levels were compared between SoJIA patients with regard to disease activity, disease courses, and other biomarkers.

**Results:**

Patients with SoJIA had significantly higher levels of IL-10 compared to patients with other febrile diseases. The serum levels of IL-10 were significantly higher in active SoJIA compared to inactive and positively correlated with known disease activity markers such as erythrocyte sedimentation rate (ESR), C-reactive protein level (CRP), ferritin (FER), and IL-6 levels. Moreover, the levels of IL-10 at diagnosis were significantly higher in SoJIA patients with a nonmonocyclic pattern than in patients with a monocyclic pattern. Compared to CRP, ESR, FER, and IL-6, IL-10 levels were superior in predicting monocyclic patients from nonmonocyclic patients.

**Conclusion:**

Compared to other febrile diseases, SoJIA patients have markedly higher levels of IL-10 which may assist with diagnosis. And a clear association of serum IL-10 levels with disease activity and disease courses in SoJIA was found. These results suggest that serum IL-10 might be a reliable clinical marker in SoJIA.

## 1. Introduction

Systemic-onset juvenile idiopathic arthritis (SoJIA) is a particular type of juvenile arthritis affecting children younger than 16 years of age, distinguished from the other subtypes of juvenile idiopathic arthritis (JIA) for its extra-articular features, such as spiking fever, evanescent rash, and serositis with elevated laboratory parameters of inflammation [1]. SoJIA is exceptional with its systemic features, which broaden the differential diagnosis to infections, malignancy, and Kawasaki disease [2]. Adult-onset Still's disease (AOSD), a systemic inflammatory disorder shared clinical similarities with SoJIA, is also known as the adult form of SoJIA [3]. Both of SoJIA and AOSD are considered to be multifactorial autoinflammatory diseases with inappropriate activation of the inflammatory cascade and disorder of inflammatory cytokine production [4]. Interleukin-10 (IL-10) is a pleomorphic cytokine with diverse phenotypic functions [5]. It is secreted by a wide variety of cells of the innate and adaptive immune system, including granulocytes, dendritic cells, macrophages, B cells, and T cell subsets [6]. In the past several years, accumulated evidences have established an important role for IL-10 in many autoimmune and inflammatory diseases [7–9]. A recent study revealed that the serum concentrations of IL-10 are increased in AOSD patients and positively correlated with disease activity [10]. It has been reported that the serum levels of IL-10 in SoJIA patients were changed in some studies [11, 12]. However, these studies only compared the expression levels of IL-10 between SoJIA patients and healthy controls; little is known about the differences of IL-10 levels between SoJIA and other fever diseases. Furthermore, whether abnormal IL-10 production is also a hallmark of disease activity in SoJIA has not been examined in detail. In the present study, we measured the serum concentration of IL-10 in patients with SoJIA and other diseases that can be easily misdiagnosed as SoJIA and analyzed the correlation between the serum levels of IL-10 and the clinical characteristics of SoJIA patients. We aimed to evaluate the utilization of serum IL-10 as a reliable clinical marker in SoJIA.

## 2. Materials and Methods

### 2.1. Patients

The serum levels of IL-10 in patients suspected or diagnosed with SoJIA have been measured before initiation of treatment as part of standard clinical care in the rheumatology department at Jiangxi Province Children's Hospital since 2016. In this noninterventional retrospective study, we retrospectively reviewed the data of 153 patients suspected or diagnosed with SoJIA that admission and follow-up at our department between January 2016 and December 2019. Medical histories and clinical and laboratory characteristics were collected from all subjects. Patients who had insufficient clinical or laboratory data were excluded. Complete data were available for 21 patients diagnosed with SoJIA, 7 patients diagnosed with systemic lupus erythematosus (SLE), 10 patients diagnosed with Kawasaki disease (KD), 6 patients diagnosed with acute lymphoblastic leukemia (ALL), and 12 patient diagnosed with severe infections (SIF). The demographic, clinical, and laboratory data for the patients are summarized in Table S1. For patients with clinically confirmed SoJIA, we also measured the serum levels of IL-10 during the follow-up, especially when disease activity changed. The therapy of hormones in combination with methotrexate (MTX) was as the basic program in patients with active disease. For some patients with severe disease manifestations, biologic agents or immunosuppressive agents are often added. In children with inactive disease, only nonsteroidal anti-inflammatory drugs were used to control SoJIA.

### 2.2. Diagnostic Criteria

The diagnosis of SoJIA was made according to the International League of Associations for Rheumatology (ILAR) Classification Criteria [13]. The diagnosis of SLE was performed in accordance with the standards developed by the American College of Rheumatology (ACR) [14]. KD was diagnosed based on the American Heart Association Guidelines [15]. The diagnosis of ALL was confirmed by a bone marrow biopsy. SIF were diagnosed by detection of pathogens in the blood, other body fluids, and tissue samples. Patients with SoJIA were further stratified into inactive SoJIA and active SoJIA. Disease activity status was assessed according to the clinical and laboratory data. The criteria for active disease were defined as follows: active arthritis, fever, rash, hepatosplenomegaly, generalized lymphadenopathy, and serositis, along with an elevated erythrocyte sedimentation rate (ESR) and C-reactive protein (CRP) levels [16]. Flare was defined as failure to control fever, a dramatic rise in inflammatory markers, worsening of other clinical features, or the progression of joint disease [17]. Inactive disease was defined according to American College of Rheumatology provisional criteria [18] as follows: no joints with active arthritis; no fever, rash, serositis, splenomegaly, or generalized lymphadenopathy attributable to JIA; no active uveitis to be defined; and ESR or CRP level within normal limits in the laboratory where tested.

### 2.3. Statistical Analysis

Descriptive data are presented as the mean ± SD or median with an interquartile range (IQR) for continuous outcomes and number and percentage (%) for categorical outcomes. Differences in data between multiple groups were analyzed using one-way ANOVA. Pairwise comparisons between groups were analyzed with Dunnett's T3 test. Differences between two groups were assessed by a 2-tailed *t*-test or Mann–Whitney *U* test, as appropriate. The correlations between IL-10 and other variables of interest were evaluated with a Pearson correlation test or Spearman's correlation test. Receiver-operating characteristic (ROC) curve analysis was performed to assess the sensitivity and specificity of IL-10, CRP, ESR, FER, and IL-6 measurement and its diagnostic and predictive utility. In all comparisons, *p* values less than 0.05 were considered statistically significant, and all *P* values corresponded to two-sided significance tests. Statistical analyses were carried out using the SPSS software version 22.0.

## 3. Results and Discussion

### 3.1. Serum IL-10 Expression Levels

**Figure 1:**
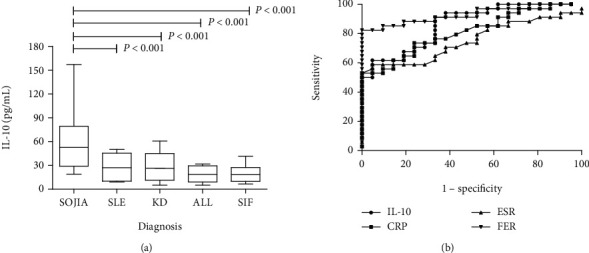
The expression of serum IL-10 and ROC curves. (a) Comparison of serum IL-10 levels in all patients. (b)ROC curve of IL-10, CRP, ESR, and FER in distinguishing SoJIA from other disease categories.

The demographic, clinical, and laboratory data for the patients are summarized in Table S1. There were some significant differences showed among the duration of fever before hospitalization, levels of serum IL-10, CRP, ESR, and FER in SoJIA compared with other febrile diseases like SLE, KD, ALL, and SIF. The serum concentration of IL-10 showed significant differences among various disease groups (*F* = 20.26, *P* < 0.001, Figure 1(a)); the SoJIA group showed a significantly higher IL-10 expression level than the SLE group (58.22 ± 33.27 vs. 28.20 ± 16.90 pg/mL, *P* < 0.001), KD group (28.97 ± 20.12 pg/mL, *P* < 0.001), ALL group (18.72 ± 10.25 pg/mL, *P* < 0.001), and SIF group (19.53 ± 11.16 pg/mL, *P* < 0.001). Receiver of characteristic curve (ROC) analysis was conducted to analyze the performances of serum IL-10, CRP, ESR, and FER in supporting the diagnosis of SoJIA compared to the other fever diseases which were frequently misdiagnosed as SoJIA. Serum IL-10 performed well with area under the curve of (AUC) 0.862 (*P* < 0.001) in differentiating SoJIA from other disease categories (Figure 1(b)). This analysis indicated an optimal cut-off to identify SoJIA was 42.23 pg/mL with a sensitivity of 91.18% and a specificity of 66.67%. The ACU of CRP, ESR, and FER are shown in Tables S2. Individual comparisons showed that serum IL-10 can effectively distinguish SoJIA from SLE, KD, ALL, or SIF. The cut-off values and their respective coordinates on the ROC curve are shown in Tables S3.

### 3.2. Serum IL-10 and Disease Activity

**Figure 2:**
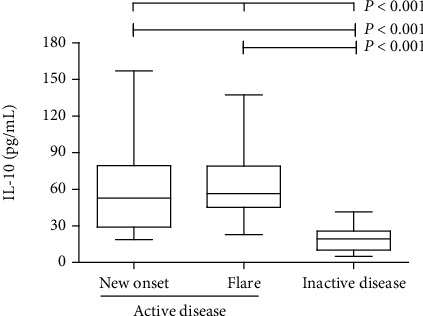
Comparison of serum IL-10 levels among patients with SoJIA with active and inactive disease activity.

SoJIA patients were stratified by disease activity. The characteristics of the SoJIA patients with active disease (new onset or flare) or inactive disease are shown in Table 1. We analyzed the association of serum IL-10 with disease activity in patients with SoJIA. As shown in Figure 2, serum IL-10 levels in patients with SoJIA were higher during the active phase (62.55 ± 28.85 pg/mL) than during the inactive phase (18.33 ± 9.10 pg/mL, *P* < 0.001). But there was no significant difference in serum IL-10 levels between patients with new-onset SoJIA and patients with disease flares (58.22 ± 33.27 vs. 64.49 ± 26.55 pg/mL; *P* = 0.408).

### 3.3. Serum IL-10 and Inflammation Markers

**Figure 3:**
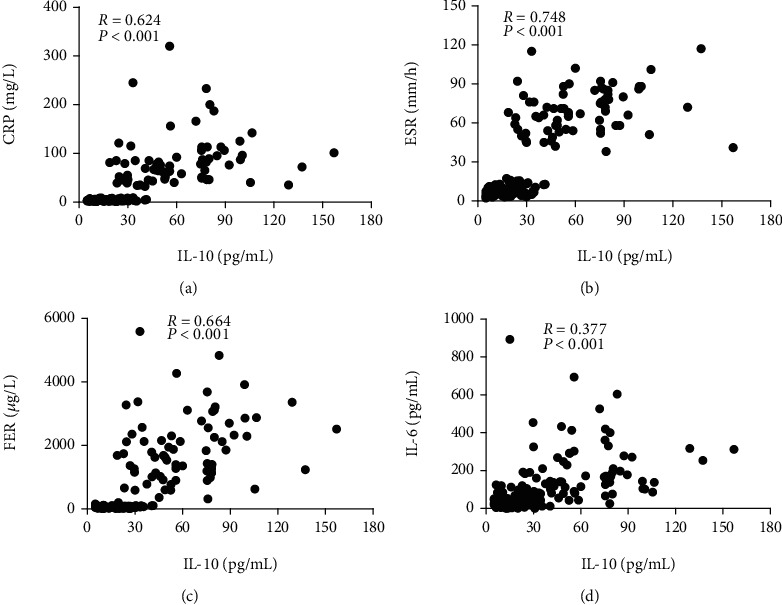
Correlation between serum IL-10 and other biomarkers. Serum IL-10 levels were positively correlated with CRP (a), ESR (b), FER (c), and IL-6 (d).

An assessment of the correlation between the serum IL-10 levels and several known markers of disease activity was performed to further explore the relationship between serum IL-10 and disease activity in SoJIA. Our results revealed that the serum IL-10 levels correlated well with the levels of CRP, ESR, FER and IL-6 (*r* = 0.624, *P* < 0.001; *r* = 0.748, *P* < 0.001; *r* = 0.664, *P* < 0.001; and *r* = 0.377, *P* < 0.001; respectively) (Figure 3).

### 3.4. Serum IL-10 and Disease Courses

**Figure 4:**
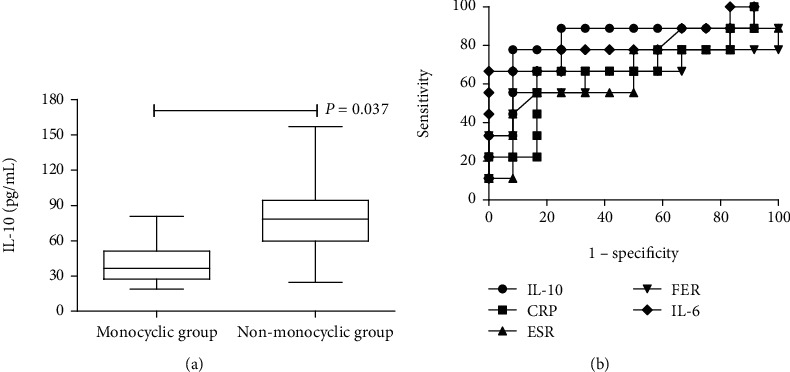
Correlation between serum IL-10 and disease courses. (a) Comparison of IL-10 levels among patients with SoJIA with monocyclic pattern and nonmonocyclic pattern. (b) ROC curves of IL-10, CRP, ESR, FER, and IL-6 for predicting disease course in SoJIA.

To assess the possible association between serum IL-10 and disease courses in patients with SoJIA, the SoJIA patients were divided into two groups. Patients who did not have a flare during the follow-up after initial remission were characterized as the monocyclic group. Patients who exhibited persistent systemic symptoms, or persistent arthritic symptoms, or both, and who experienced disease flares during the follow-up were characterized as the nonmonocyclic group. The characteristics of these two groups of patients are shown in Table 2. We analyzed the expression levels of serum IL-10 in these patients at diagnosis. Our results revealed that the serum IL-10 levels at diagnosis were significantly higher in nonmonocyclic group patients than in monocyclic group patients (80.36 ± 36.73 vs. 41.61 ± 18.14 pg/mL, *P* = 0.005) (Figure 4(a)). To determine the predictive value of serum IL-10 for disease courses, the ROC analysis was performed. The results demonstrated a better predictive accuracy for IL-10 than for CRP, ESR, FER, and IL-6 to detect a nonmonocyclic pattern. The ACU was 0.824 for IL-10, 0.667 for CRP, 0.664 for ESR, 0.648 for FER, and 0.810 for IL-6 (Figure 4(b)). At a cut-off value of 65.75 ng/mL, serum IL-10 was 77.78% sensitive and 91.67% specific in differentiating between monocyclic patients and nonmonocyclic patients (*P* = 0.013) (Table S4).

## 4. Discussion

IL-10 is a pleiotropic cytokine produced by a wide variety of cells. It has been implicated in several major diseases including cancer, rheumatic disease, and infectious disease [5]. The levels of IL-10 are increased in many inflammatory diseases such as SLE, RA, and SSc. Recently, Sun et al. found that the serum concentrations of IL-10 are increased in AOSD patients and positively correlated with disease activity [10]. SoJIA shared clinical similarities with AOSD, despite the age of onset being younger than 16 years [19]; substantial advances have been made to confirm the homology between SoJIA and AOSD [20, 21]. However, the levels of IL-10 in SoJIA patients are still controversial. Cheng et al. revealed that the serum levels of IL-10 were increased in SoJIA patients compared with those in healthy controls [22]. Guo et al. found that the levels of IL-10 in children with SoJIA were lower than those in healthy children group [12]. Thus, to address this issue, we examined the serum levels of IL-10 in SoJIA patients with a highly sensitive electrochemiluminescence assay.

In this study, we compared the expression of serum IL-10 between SoJIA patients and other febrile diseases which were often misdiagnosed as SoJIA. The serum levels of IL-10 were significantly increased in SoJIA patients than in patients with SLE, KD, ALL, or SIF. The diagnosis of SoJIA relies on clinical signs and symptoms. None of the specific biomarker had been established to date. Since many of the presenting symptoms are nonspecific, diagnosing SoJIA can be extremely challenging if typical arthritis is lacking. It is crucial to exclude other differential diagnoses such as infection, malignancy, other autoimmune disease, and autoinflammatory conditions [23]. Interestingly enough, we found that IL-10 would be a useful diagnostic marker for SoJIA. It can effectively distinguish SoJIA from other non-SoJIA causes of fever such as SLE, KD, ALL, or SIF. In addition, our results showed that the serum IL-10 levels in patients with SoJIA were higher during the active phase than during the inactive phase. Furthermore, serum IL-10 levels in SoJIA positively correlated with some nonspecific markers of inflammation such as ESR, CRP, FER, and a known disease activity marker IL-6 [23, 24]. Our results were similar with a study conducted in patients with AOSD, which found that the serum IL-10 was positively correlated with disease activity and inflammatory markers [10]. These findings strongly indicate that IL-10 has an important role in pathogenesis of SoJIA and can be a good marker for monitoring disease activity in SoJIA.

In addition to the diagnostic challenges associated with fevers of unknown origin and fever in children with SoJIA, prognostic challenges are prominent in SoJIA. SoJIA can be distinguished into three types according to the clinical courses: monocyclic, polycyclic, and persistent courses [25]. The monocyclic pattern, which is the most benign form, is characterized by a single illness episode attributed to SoJIA and then essentially disease remission. In the polycyclic pattern, the patient experiences recurrent attacks. Other patients exhibit a persistent pattern with unremitting disease manifested by persistent systemic, or persistent arthritic symptoms, or both [26]. The prognosis of SoJIA is largely dependent upon the disease course. Long-term outcome studies have indicated that patients with SoJIA with polycyclic or persistent disease course display greater disability, weakness, and disease damage than those with monocyclic disease course [2]. Therefore, detecting disease course in its early stage may be essential to the management of SoJIA. However, guidance on establishing an early detection of disease course in SoJIA is limited. Recently, Zhao et al. found that early production of IL-10 was significantly associated with disease severity in COVID-19, a global and life-threatening pandemic which is characterized by high levels of cytokines such as IL-1, IL-2, IL-4, IL-6, and IL-10 in infected individuals [27, 28]. Furthermore, Han et al. found that IL-6 and IL-10 can be used as predictors for fast diagnosis of COVID-19 patients with higher risk of disease deterioration [29].

Our data show that the serum IL-10 levels at diagnosis were significantly higher in SoJIA patients with polycyclic or persistent disease course than those patients with monocyclic disease course. Further, with our present results, we reveal a better predictive accuracy for IL-10 than for CRP, ESR, FER, and IL-6 to detect a nonmonocyclic pattern, which means that IL-10 can be used to discriminate SoJIA patients at diagnosis as prone to a monocyclic disease course or other disease courses. The additional ability to detect disease courses with a higher sensitivity than ESR, FER, and IL-6 makes the assessment of serum IL-10 levels a valuable tool in the clinical assessment of patients with SoJIA.

It is well known that IL-10 mainly acts as an anti-inflammatory cytokine in many diseases [5]. Interestingly, our data confirmed the increased serum levels of IL-10 in patients with SoJIA and demonstrated a possible role of IL-10 as a disease biomarker in SoJIA. A recent study revealed that serum levels of IL-37, which functions as a natural suppressor of inflammatory and immune responses, were increased in patients with AOSD and associated with AOSD disease activity [30]. Moreover, their data show that serum IL-37 levels were positively correlated with IL-10. They speculate that inflammation signaling not only exacerbated the inflammatory response in the pathogenesis of AOSD but also promoted the expression of anti-inflammatory cytokines such as IL-37 and IL-10 to limit excessive inflammation in AOSD [30, 31]. According to the above results, we hypothesize that there exists a similar principle in IL-10 and a feedback loop from proinflammatory cytokines to the upregulation of anti-inflammatory cytokines in SoJIA.

This study has several limitations. Small sample was the first limitation due to rarity of SoJIA. Another limitation was the insufficiency of follow-up time. As the definition of disease course is strongly based on the follow-up time, we would not know if some of the monophasic patients might flare in the future, although all monophasic patients were followed at least several years in our center without any flare. Then, the long-term outcomes of patients might be different from the visit in our center in terms of disease courses. Finally, it is a single-center, retrospective cohort study. Therefore, multicenter studies with larger cohorts will be necessary to further verify our findings.

To summarize, our study showed that upregulation of IL-10 was positively correlated with SoJIA disease activity, and the levels of IL-10 at diagnosis might predict the disease courses of SoJIA. Monitoring of serum IL-10 levels may be useful for assessing SoJIA disease activity and predicting SoJIA disease courses, although further large studies with a greater number of SoJIA patients are necessary to confirm the usefulness of serum IL-10 in daily clinical practice.

## Figures and Tables

**Table 1:** Characteristics of the patients with active SoJIA or inactive disease.

Characteristics	Inactive disease	Active disease	
		New onset	Flare
Patients (*n*)	21	21	19
Samples (*n*)	87	21	47
IL-10 (pg/mL)	18.33 ± 9.10	58.22 ± 33.27	64.49 ± 26.55
CRP (mg/mL)	3.78 ± 1.87	115.86 ± 78.61	73.64 ± 29.51
ESR (mm/h)	8.25 ± 3.51	71.14 ± 19.99	68.91 ± 16.38
FER (*μ*g/L)	59.42 ± 33.41	2095.71 ± 1281.48	1881.64 ± 999.12
IL-6 (pg/mL)	53.96 ± 96.81	259.68 ± 302.27	197.35 ± 115.38

**Table 2:** Characteristics of SoJIA patients with monocyclic pattern or nonmonocyclic pattern.

Characteristics	Monocyclic group	Nonmonocyclic group	*t*	*P*
Patients (*n*)	12	9		
IL-10 (pg/mL)	41.61 ± 18.14	80.36 ± 36.73	-3.190	0.005
CRP (mg/mL)	94.00 ± 60.12	145.00 ± 93.85	-1.518	0.145
ESR (mm/h)	67.00 ± 16.85	76.67 ± 23.43	-1.102	0.284
FER (*μ*g/L)	1712.25 ± 654.62	2607.00 ± 1732.52	-1.473	0.172
IL-6 (pg/mL)	116.69 ± 59.63	450.33 ± 389.83	-2.545	0.034

## Data Availability

Data was available on request.

## Supplementary Materials

Supplementary Materials Table S1: clinical characteristics of patients with SoJIA and other fever diseases. Table S2: analysis of the ROC curves of IL-10, CRP, ESR, and FER in diagnosing SoJIA. Table S3: analysis of the ROC curves of IL-10. Table S4: analysis of the ROC curves of IL-10, CRP, ESR, FER, and IL-6 in predicting disease course.
