# Supplementary material for: The Association of Serum IL-10 Levels with the Disease Activity in Systemic-Onset Juvenile Idiopathic Arthritis Patients
Source: Mediators Inflamm. 2021 Mar 22;2021:6650928. doi: 10.1155/2021/6650928 (PMC8007368; doi:10.1155/2021/6650928)
Supplement: Supplementary Materials — Table S1: clinical characteristics of patients with SoJIA and other fever diseases. Table S2: analysis of the ROC curves of IL-10, CRP, ESR, and FER in diagnosing SoJIA. Table S3: analysis of the ROC curves of IL-10. Table S4: analysis of the ROC curves of IL-10, CRP, ESR, FER, and IL-6 in predicting disease course. [file 6650928.f1.doc]

Supplementary Table S1. Clinical characteristics of patients with SoJIA and other fever diseases

| Characteristics | SoJIA | SLE | KD | ALL | SIF | F/ χ2 | P value |
| --- | --- | --- | --- | --- | --- | --- | --- |
| Patients, n | 21 | 7 | 10 | 6 | 12 |  |  |
| Male/female | 13/8 | 5/2 | 6/4 | 3/3 | 5/7 | 2.181 | 0.736 |
| Age, years | 3.6 ±1.1 | 4.5 ±2.3 | 2.3 ±1.5 | 3.8 ±1.6 | 2.7 ±2.1 | 1.24 | 0.155 |
| Duration of fever before hospitalization, days | 9.24±2.79 | 8.71±1.50 | 5.20±1.23* | 8.17±2.14 | 7.00±1.90* | 6.44 | <0.001 |
| IL-10, pg/mL | 58.22±33.27 | 28.20± 16.90* | 28.97±20.12* | 18.72±10.25* | 19.53±11.16* | 20.26 | <0.001 |
| CRP, mg/ mL | 115.86±78.61 | 17.29±11.97* | 58.00±28.93* | 17.50±9.61* | 83.09±55.40 | 6.27 | <0.001 |
| ESR, mm/h | 71.14±19.99 | 61.71±37.35 | 69.10±27.34 | 48.33±23.39* | 14.18±6.39* | 12.55 | <0.001 |
| FER, μg/L | 2095.71±1281.48 | 793.29±764.40* | 400.00±364.28* | 608.00±416.42* | 160.55±60.63* | 14.30 | <0.001 |

* Significant differences compared with SoJIA

Supplementary Table S2.Analysis of the ROC curves of IL-10, CRP, ESR and FER in diagnosing SoJIA.

|  | Cutoff value | ROC area | Sensitivity(%) | Specificity(%) | P value |
| --- | --- | --- | --- | --- | --- |
| IL-10, pg/mL | 42.23 | 0.862 | 91.18 | 66.67 | <0.001 |
| CRP, mg/ mL | 63.00 | 0.810 | 73.53 | 76.19 | <0.001 |
| ESR, mm/h | 42.00 | 0.752 | 58.88 | 95.24 | <0.001 |
| FER, μg/L | 549 | 0.928 | 82.35 | 95.24 | <0.001 |

Supplementary Table S3.Analysis of the ROC curves of IL-10

|  | Cutoff value | ROC area | Sensitivity(%) | Specificity(%) | P value |
| --- | --- | --- | --- | --- | --- |
| Compared to the SLE group | 51.37 | 0.803 | 100 | 52.38 | 0.018 |
| Compared to the KD group | 41.63 | 0.776 | 80.00 | 66.67 | 0.014 |
| Compared to the ALL group | 32.33 | 0.905 | 100 | 71.43 | 0.003 |
| Compared to the SIF group | 23.64 | 0.913 | 72.73 | 95.24 | <0.001 |

Supplementary Table S4.Analysis of the ROC curves of IL-10, CRP, ESR, FER and IL-6 in predicting disease course.

|  | Cutoff value | ROC area | Sensitivity(%) | Specificity(%) | P value |
| --- | --- | --- | --- | --- | --- |
| IL-10, pg/mL | 65.75 | 0.824 | 77.78 | 91.67 | 0.013 |
| CRP, mg/ mL | 96.50 | 0.667 | 66.67 | 83.33 | 0.201 |
| ESR, mm/h | 83.00 | 0.644 | 55.56 | 83.33 | 0.271 |
| FER, μg/L | 2432 | 0.648 | 55.56 | 91.67 | 0.256 |
| IL-6, pg/ mL | 200 | 0.810 | 66.67 | 91.67 | 0.017 |
